# A Bioactive Degradable Composite Bone Cement Based on Calcium Sulfate and Magnesium Polyphosphate

**DOI:** 10.3390/ma17081861

**Published:** 2024-04-18

**Authors:** Suping Peng, Xinyue Yang, Wangcai Zou, Xiaolu Chen, Hao Deng, Qiyi Zhang, Yonggang Yan

**Affiliations:** 1 School of Chemical Engineering, Sichuan University, Chengdu 610065, China; 2 College of Physics, Sichuan University, Chengdu 610065, China

**Keywords:** bone defects, calcium sulfate, magnesium polyphosphate, bone cement

## Abstract

Calcium sulfate bone cement (CSC) is extensively used as a bone repair material due to its ability to self-solidify, degradability, and osteogenic ability. However, the fast degradation, low mechanical strength, and insufficient biological activity limit its application. This study used magnesium polyphosphate (MPP) and constructed a composite bone cement composed of calcium sulfate (CS), MPP, tricalcium silicate (C_3_S), and plasticizer hydroxypropyl methylcellulose (HPMC). The optimized CS/MPP/C_3_S composite bone cement has a suitable setting time of approximately 15.0 min, a compressive strength of 26.6 MPa, and an injectability of about 93%. The CS/MPP/C_3_S composite bone cement has excellent biocompatibility and osteogenic capabilities; our results showed that cell proliferation is up to 114% compared with the control after 5 days. After 14 days, the expression levels of osteogenic-related genes, including Runx2, BMP2, OCN, OPN, and COL-1, are about 1.8, 2.8, 2.5, 2.2, and 2.2 times higher than those of the control, respectively, while the alkaline phosphatase activity is about 1.7 times higher. Therefore, the CS/MPP/C_3_S composite bone cement overcomes the limitations of CSC and has more effective potential in bone repair.

## 1. Introduction

Bone defects resulting from trauma, infection, and tumors are the main reasons for bone nonunion or delayed bone healing, which in turn suffer from muscle atrophy and limb dysfunction [1,2,3,4]. Conventional natural bone repair materials used in clinics pose risks of secondary injury, infection, and immune response [5,6]. Recently, with advancements in materials science and increasing clinical demands, artificial bone repair materials have emerged as promising alternatives for bone defect reconstruction. Among these, bone cement stands out for its exceptional injectability, osteogenic capacity, and ability to self-solidify under physiological circumstance [7,8]. As an ideal active bone repair material, it promotes osteogenic growth and gradually degrades during the process of osteogenic growth, eventually being entirely substituted by the new bone. Among the materials with self-setting property, calcium sulfate bone cement (CSC) has gained recognition as a safe and effective option, boasting excellent biocompatibility, complete degradation, and in vivo and in situ self-curing. Nevertheless, some shortcomings, including rapid degradation, low mechanical strength, and insufficient biological activity, limit its application [9,10,11]. A a result, a lot of research has been conducted on composite bone cement based on calcium sulfate. 

Tricalcium silicate (C_3_S), a calcium silicate-based biomaterial, serves as another self-setting component in bone cement. Its characteristics include excellent biodegradability and biological activity, and it generates calcium hydroxide during hydration, which can also compensate for the acidity and rapid degradation of CSC [12,13,14,15]. More importantly, Si, a significant element in the human body, can enhance the expression of type I collagen and promote the secretion of extracellular signal-regulated protein kinases [16]. An appropriate concentration of Si can inhibit the generation of osteoclasts [17], enhance the in vitro activity of osteoblasts [16], promote osteoblast differentiation and angiogenesis [18], and accelerate new bone deposition.

It is well known that the inorganic component of bone is calcium phosphate salts, thus much literature has studied the source of phosphate ions in the process of bone mineralization to form hydroxyapatite (HA) [19]. Earlier, organic phosphates, particularly β-glycerophosphate, were proposed as a source of phosphate for mineralization. However, such compounds undergo rapid dephosphorylation in the extracellular space and are not available in sufficient quantities to serve as a source of bone formation. Therefore, inorganic polyphosphate (PolyP) was proposed as a source of phosphate and became widely recognized [20,21]. In the process of bone mineralization, PolyP, renowned for its excellent biocompatibility, biological activity, and biodegradability, not only serves as a phosphate source of hydroxyapatite, but releases chemical energy during enzymatic hydrolysis by alkaline phosphatase (ALP) [22]. Within bone and cartilage tissues, PolyP acts as a regulator of gene expression, promotes the acceleration of osteoblast mineralization, and inhibits the differentiation of osteoblast precursors into osteoclasts [23,24,25,26]. Additionally, PolyP can form crosslinks with Mg^2+^, further promoting bone formation through ionic bonds at neutral pH [27,28,29]. Therefore, MPP holds promise in enhancing the bioactivity of CSC and fostering bone formation. Moreover, inspired by the composition of natural bone tissue, organic polymers are considered as candidates to enhance the mechanical properties of bone cement [14,15,30,31]. Hydroxypropyl methylcellulose (HPMC), possessing good biocompatibility and biodegradability, improves the mechanical properties, injectability, and collapsibility of bone cement [32,33,34]. Thus, MPP, C_3_S, and HPMC are expected to synergistically contribute to CSC, achieving appropriate biomechanical properties, degradation rate, and pH value, along with fostering good biological activity and promoting bone differentiation. This advancement aims to improve the applicability of CSC as a bone-repair material.

**Figure 1:**
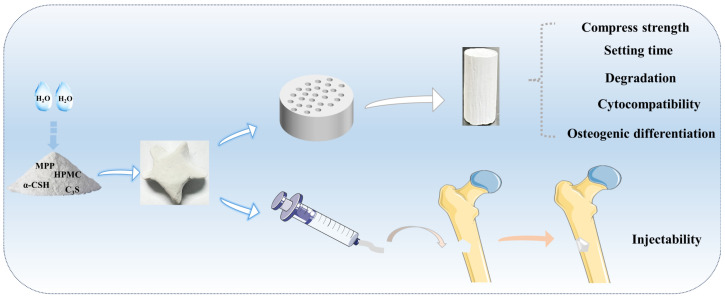
Diagram of the preparation and evaluation of composite bone cement.

Herein, a degradable CS/MPP/C_3_S composite bone cement was prepared by incorporating MPP, C_3_S, and HPMC. CS and C_3_S serve as prerequisites for the self-solidification of the composite bone cement. MPP and C_3_S can synergistically regulate the degradation rate and microenvironment of degradation. Additionally, HPMC is able to enhance the mechanical properties, injectability, and collapsibility of the composite bone cement. Subsequently, the physicochemical and in vitro biological characteristics of CS/MPP/C_3_S composite bone cement were further evaluated. A schematic diagram of the preparation of bone cement and its injectability is shown in Figure 1.

## 2. Materials and Methods

### 2.1. Materials

α-calcium sulfate hemihydrate (α-CSH, 97%) was supplied by Huzhou ZHAN WANG Pharmaceutical Industry (Huzhou, China); NaH_2_PO_4_·2H_2_O (99%, AR), KH_2_PO_4_ (99%, AR), Na_2_HPO_4_·12H_2_O (99%, AR), NaCl (99%, AR), calcium acetate, and KCl (99%, AR) were purchased from Chengdu Kelong Chemical Co., Ltd. (Chengdu, China); Gallic acid (99%, AR) and KOH (98%, AR) were purchased from Chron Chemicals (Chengdu, China); MgCl_2_·6H_2_O (98%, AR) and Hypromellose (E60, 20,000 mPa·s) were obtained from ADAMAS-BETA Reagent (Shanghai, China); and tricalcium silicate was supplied by Kunshan Chinese Technology New Materials Co., Ltd. (Kunshan, China) A Cell Counting Kit-8 (CCK-8) was purchased from Nanjing Keygen Biotech (Nanjing, China), and an alkaline phosphatase (ALP) activity assay kit was purchased from Nanjing Jiancheng Bioengineering Institute (Nanjing, China). Alizarin Red S solution was obtained from Cyagen Biosciences (Guangzhou, China). All water (18.25 MΩ·cm) was obtained from an ultrapure water system (RES. J Scientific Instruments Co., Ltd., Kyoto, Japan).

### 2.2. Preparation and Optimizing of CS/MPP/C_3_S Composite Bone Cement

MPP was fabricated referring to the literature [28,35]. The mass ratio of α-CSH, MPP, and C_3_S was optimized, and a final ratio of 87:10:3 in the preparation of CS/MPP/C_3_S composite bone cement was obtained. Then the plasticizer was added; the specific components were listed in Table S2. Specifically, each component in proportion was mixed evenly through ball milling for 3 h. The cylindrical bone cement was obtained by evenly mixing ultrapure water and bone cement powder (liquid to solid ratio: 0.25 mL/g), and injecting the mixture into a polytetrafluoroethylene (PTFE) mold. The bone cement was de-molded after solidification and stored at room temperature for further testing.

### 2.3. Characterization of CS/MPP/C_3_S Composite Bone Cement

The structure and composition of CS/MPP/C_3_S composite bone cement were characterized by X-ray diffraction (XRD, Panalytical, X’Pert Pro MPD DY129), Fourier transform infrared spectroscopy (FT-IR, Bruker, Billerica, MA, USA, invenio r), and scanning electron microscopy (SEM, Thermo Scientific (Waltham, MA, USA), Helios 5 CX).

The solidification time of CS/MPP/C_3_S composite bone cement was tested with a Vicat apparatus according to the ISO 9597-2008 standard [36]. The cylindrical samples with *Φ*6 × 4 mm were measured, and three replicates were performed for each group of samples (*n* = 3).

For the compressive test, cylindrical samples with *Φ*6 × 12 mm were measured after CS/MPP/C_3_S composite bone cement was cured for 3 days. The compressive strength was measured with a universal testing machine (Instron, Norwood, MA, USA, 5567) with a loading rate of 1 mm/min and a sensor load of 20 KV, and six replicates were performed for each group of samples (*n* = 6). The injectability and anti-collapsibility were tested according to the method in reference [37] (*n* = 3).

### 2.4. Degradation In Vitro

For evaluation of degradation, the cylindrical samples with *Φ*6 × 12 mm were immersed in phosphate buffer solution (PBS; pH 7.40), the cements were immersed in PBS solution (surface area to solution volume ratio of 0.1 cm^2^/mL) and placed in an oscillating water bath (37 °C, 80 rpm/min); the pH of the cement soaking solution in each group was measured with a pH meter at suitable time points. The PBS solution was replaced after each test in the first week, and then replaced every 7 days. Additionally, the concentrations of Ca^2+^ and Mg^2+^ in the degradation solution were also tested by atomic absorption spectroscopy (VARIAN, SpectrAA 220FS/220Z). At each test time point, the samples were taken out and dried at 60 °C for 24 h to calculate the weight loss ratio. The weight loss ratio was calculated according to the Equation (1) (*n* = 3):

(1) $$\mathrm{Weight}\;\mathrm{loss}\;(\%)=\frac{W_{0}-W_{t}}{W_{0}}\times 100\%$$

where *W*_0_ is the initial mass of the bone cement, *W*_t_ is the mass of the bone cement after degradation. Three parallel samples were used for each test.

### 2.5. Cytocompatibility

The extract was prepared according to the ISO 10993-12 standard [38]. Specifically, the samples were immersed in minimum essential medium alpha (MEM-α) at a concentration of 200 mg/mL, and incubated in a CO_2_ incubator (37 °C, 5% CO_2_, 100% humidity) for 24 h. Subsequently, the extracts were centrifuged and filtered with a 0.22 μm sterile filter membrane. Then 10 vol% fetal bovine serum (FBS) was added to the filtered extract. The resulting solution was diluted to the required concentration with the complete culture medium containing 10 vol% FBS, 1 vol% penicillin–streptomycin solution, and 89% MEM-α.

Cell proliferation: Mouse embryonic osteoblast cells (MC3T3; Cell Bank of the Chinese Academy of Sciences, Shanghai, China) were seeded at a density of 1.5 × 10^3^ cells per well (*n* = 6) in the 96-well culture plates, and incubated in a CO_2_ incubator. After the cells adhered to the bottom of the wall (~24 h), the culture medium was replaced by material extracts, and renewed every 48 h. Subsequently, 10 vol% of Cell Counting Kit-8 (CCK-8) solution was added to each well after 1, 3, and 5 days, and the optical density (OD) value was measured at 450 nm by the microplate reader.

Fluorescence staining: MC3T3 were seeded at a density of 1.5 × 10^4^ cells per well (*n* = 3) in a 24-well plate and cultured with extracts for 3 days. Subsequently, the cells were washed with PBS, fixed with 4% glutaraldehyde solution for 15 min, and permeabilized with 0.5 vol% Triton^TM^ X-100 for 5 min, and washed with PBS again 3 times. The cytoplasm of the cells was stained with Alexa Fluor-555-labeled phalloidin, and then 4′, 6-amidine-2-phenylindole dihydrochloride (DAPI) was added to re-stain the nuclei. Finally, the cytoskeleton was visualized under a fluorescence microscope.

### 2.6. Osteogenic Differentiation

The material extract was prepared as above. Mouse bone marrow-derived mesenchymal stem cells (mBMSCs) were cultured using an osteogenic induction medium. The cell culture process was the same as above, but the difference was that MEM-α was replaced with Dulbecco’s modified Eagle’s medium (DMEM). In addition, the osteogenic induction medium was obtained by adding 50 μg/mL of ascorbic acid, 100 nM of dexamethasone, and 10 mM of β-sodium glycerophosphate to the complete culture medium. The mBMSCs were seeded at a density of 1.5 × 10^4^ cells per well (*n* = 6) in a 24-well plate. ALP activity of the supernatant was measured after 7 and 14 days according to the ALP activity assay kit. On days 14 and 21, cells were fixed with 4% glutaraldehyde solution, and calcium nodules were stained with Alizarin Red, recording the result by taking photos. Then, the staining solution was removed, and 10% cetylpyridine chloride was introduced. The OD value at 620 nm was measured using a microplate reader to obtain semi-quantitative calcium deposition. Additionally, mBMSCs were seeded at a density of 1.5 × 10^5^ cells per well (*n* = 3) in a 6-well plate. The total RNA from the cells was extracted and the concentration was determined through nucleic acid analysis after 14 days. Osteogenesis-related genes (BMP2, OCN, Runx2, OPN, and COL1) were detected using a q-PCR fluorescence analysis system (Gen Bio Medical FQD-96C, Richmond, BC, Canada), and the gene expression ratio was calculated using the 2^−ΔΔCT^ method with the reference gene β-actin for normalization. The RT-PCR primer sequences are listed in Table 1 [39,40].

### 2.7. Statistical Methods

Data are presented as mean ± SD, and one-way analysis of variance (ANOVA) was used to compare the data of different groups. Values of * *p* < 0.05, ** *p* < 0.01, and *** *p* ≤ 0.001 were considered statistically significant.

## 3. Results and Discussion

### 3.1. Optimization of Bone Cement Components

In the exploration process of the experiment, we found that the hydration of CSH was affected by MPP, resulting in a longer solidification time of bone cement. Moreover, both CS and MPP produced acidic substances during the degradation process. Therefore, C_3_S, a component of self-setting bone cement, was added to CS/MPP/C_3_S composite bone cement to address this issue. On the one hand, C_3_S generated Ca (OH)_2_ during the hydration process, which could neutralize the acidic substances produced by the degradation of MPP and CS. On the other hand, C_3_S released a large amount of heat during the hydration process, which may promote the hydration reaction of CSH, thereby regulating the solidification time of CS/MPP/C_3_S composite bone cement. However, the high content of C_3_S caused the temperature to be too high during the solidification of CS/MPP/C_3_S composite bone cement. In order to comprehensively adjust the curing time, curing temperature, and degradation pH of composite bone cement, the optimal mass ratio for α-CSH, MPP, and C_3_S was set at 87:10:3.

**Figure 2:**
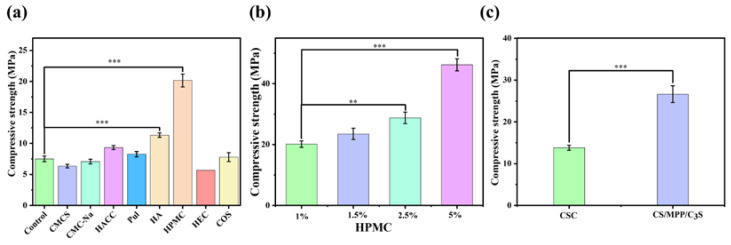
Optimization of bone cement components. (**a**) Compressive strength of bone cement with different plasticizers. (**b**) Compressive strength of CS/MPP/C_3_S composite bone cement with different contents of HPMC. (**c**) Compressive strength of CSC and CS/MPP/C_3_S composite bone cement (** *p* < 0.01, and *** *p* ≤ 0.001).

In addition, organic compounds with good biocompatibility were added as a plasticizer to improve the compressive strength of composite bone cement. Firstly, carboxymethyl chitosan (CMCS), carboxymethyl cellulose sodium (CMC-Na), chitosan quaternary ammonium salt (HACC), pullulan polysaccharide (Pul), hyaluronic acid (HA), hydroxypropyl methylcellulose (HPMC), hydroxyethyl cellulose (HEC), and chitosan oligosaccharides (COS), which accounted for 1% of the total mass of the inorganic phase, were respectively added to composite bone cement. Then, the compressive strength was tested to select a suitable plasticizer. As shown in Figure 2a, the compressive strength of bone cement with CMCS, CMC-Na, HACC, Pul, HA, HPMC, HEC, and COS were 6.3 MPa, 7.1 MPa, 9.3 MPa, 8.3 MPa, 11.3 MPa, 20.2 MPa, 7.4 MPa, and 5.4 MPa, respectively. Clearly, the addition of HPMC greatly promoted the compressive strength of bone cement.

Therefore, HPMC was selected as the plasticizer for CS/MPP/C_3_S composite bone cement. Furthermore, the effect of HPMC content was investigated. We added 1%, 1.5%, 2.5%, and 5% of HPMC to composite bone cement, and the compressive strength was measured to screen for the appropriate content. As shown in Figure 2b, with the increase in HPMC content, the compressive strength of CS/MPP/C_3_S composite bone cement gradually increased. The compressive strength of CS/MPP/C_3_S composite bone cement with 1%, 1.5%, 2.5%, and 5% HPMC added was 20.2 MPa, 23.5 MPa, 28.8 MPa, and 46.2 MPa, respectively. However, an increase of HPMC may lead to an extension of setting time and a slowdown in the degradation rate. Therefore, the addition of HPMC was selected at 2%. As shown in Figure 2c, the compressive strengths of CSC and CS/MPP/C_3_S composite bone cement were 13.8 MPa and 26.6 MPa, respectively, indicating that the compressive strength of CSC was effectively improved.

### 3.2. Characterization and Properties of Bone Cement

**Figure 3:**
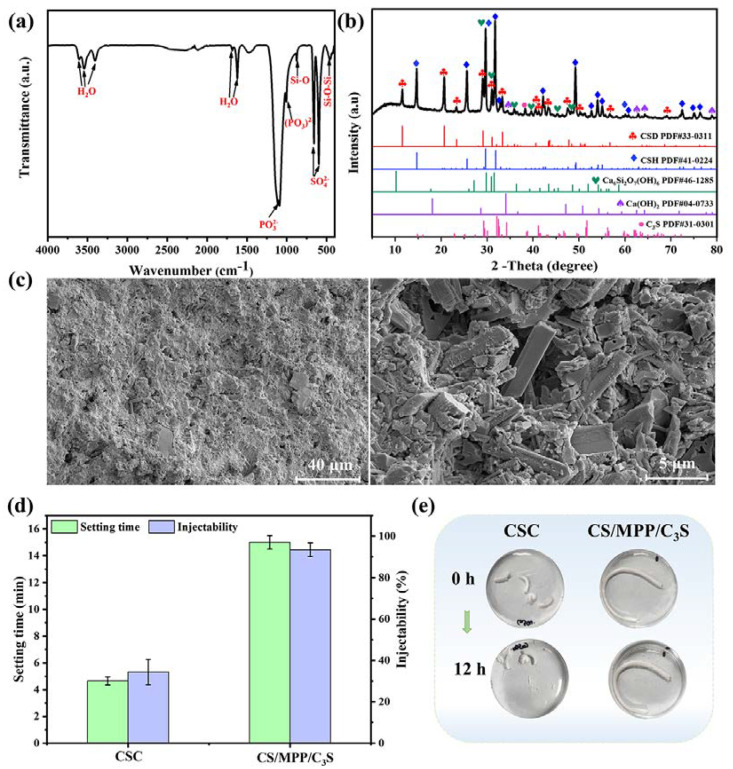
Characterization of CS/MPP/C_3_S composite bone cement. (**a**) FT-IR spectrum. (**b**) XRD pattern. (**c**) Morphologies of the cross-section. (**d**) The setting time and quantitative results of injectability of bone cement. (**e**) Injectability and anti-washout of bone cement.

The FT-IR spectrum of bone cement is shown in Figure 3a. The absorptions at 3600 cm^−1^, 3540 cm^−1^, 1685 cm^−1^, and 1620 cm^−1^ were attributed to the presence of free water in the material [41]. Additionally, the absorption peaks at 656 cm^−1^ and 590 cm^−1^ were assigned to SO_4_^2−^ of CS, while peaks at 1086 cm^−1^ and 998 cm^−1^ corresponded to PO_3_^2−^ of MPP. The peaks at 873 cm^−1^ and 447 cm^−1^ were attributed to Si-O and O-Si-O in the hydrated products of C_3_S, respectively.

The XRD of bone cement is presented in Figure 3b. After solidification, the bone cement mainly contained calcium sulfate dihydrate (CSD), α-CSH, Ca_6_Si_2_O_7_(OH)_6_, Ca(OH)_2_, and C_3_S. Among these, CSD was the hydration product of α-CSH; Ca_6_Si_2_O_7_(OH)_6_ and Ca(OH)_2_ were the hydration products of C_3_S. In addition, due to the amorphous nature of MPP and HPMC in bone cement, as well as their relatively low content compared to CS, their impact on the overall crystallinity of bone cement was relatively small. Therefore, the crystallinity of bone cement in the XRD pattern was almost unaffected.

The cross-sectional morphologies of bone cement are shown in Figure 3c. Although bone cement is a dense solid, micropores are still distributed throughout composite bone cement. As a degradable material, the micropores not only provide space for cell migration and nutrient transport in the bone repair process, but also facilitate the absorption of ions necessary for mineralization and local accumulation.

The setting time of bone cement is usually required to be within 20 min for clinical surgery. As shown in Figure 3d, CSC had a very short setting time of 4.7 ± 0.3 min, which might cause CSC to solidify before reaching a plastic state, thus preventing it from completely filling the bone defect area. The solidification time of CS/MPP/C_3_S composite bone cement was 15.0 ± 0.5 min, indicating that the solidification time of CSC could be effectively adjusted and prolonged by incorporating MPP, C_3_S, and HPMC.

Next, the injectability and anti-washout properties of bone cement were evaluated, as shown in Figure 3d,e. CSC solidified rapidly in the syringe, hindering continuous extrusion. The extrudate was uneven under pressure, with a wet extruded portion and a dry portion remaining in the syringe. The mass of the extruded part only accounted for about 34%, indicating poor injectability. Conversely, CS/MPP/C_3_S composite bone cement exhibited favorable injectability, with uniform and continuous extrusion through the syringe, accounting for 93% of the extruded mass. Subsequently, the extruded strips for testing injectability were soaked in ultrapure water for 12 h to assess their anti-washout performance. After 12 h, both CSC and CS/MPP/C_3_S composite bone cement could maintain their shapes, but there was minor particle shedding on the surface of CSC. These results demonstrated that CS/MPP/C_3_S composite bone cement exhibited excellent anti-washout performance.

### 3.3. Degradation of Bone Cement In Vitro

**Figure 4:**
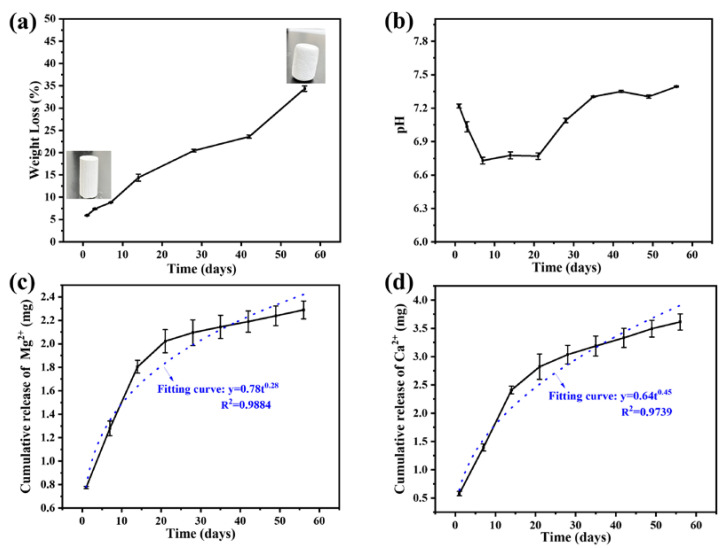
Degradation of CS/MPP/C_3_S composite bone cement in vitro. (**a**) Weight loss ratio curve (inset photos show before and after degradation) and (**b**) the variation in the pH value in PBS. (**c**) Cumulative release of Mg^2+^ and (**d**) cumulative release of Ca^2+^ in PBS.

The degradation of bone cement might provide the components and form the space for new bone growth. However, the excessive degradation did not match the rate of new bone formation, and also significantly reduced the mechanical strength of bone cement. The weight loss ratios of CS/MPP/C_3_S composite bone cement soaked in PBS for different durations are shown in Figure 4a. The CS/MPP/C_3_S composite bone cement gradually degraded in PBS over time, and the weight loss ratio was recorded to be about 35% after 56 days, which was lower than the weight loss ratio of CSC in the reported research [11], indicating the degradation rate of CSC was effectively slowed down by adding MPP, C_3_S, and HPMC. Furthermore, CSC released acidic products during degradation, reducing the pH value of the surrounding environment. The pH evolution during the degradation process of bone cement is shown in Figure 4b. In the initial stages of degradation, a mildly acidic environment was observed in CS/MPP/C_3_S composite bone cement, but the pH values remained above 6.5. This was because C_3_S in composite bone cement produced Ca (OH)_2_ during hydration, thus neutralizing the acidic products generated by CS during degradation and adjusting the pH value of the surrounding environment. The release of Mg^2+^ and Ca^2+^ during the degradation process of bone cement is shown in Figure 4c and Figure 4d, respectively. During the degradation process, the release trends of Ca^2+^ and Mg^2+^ were basically consistent with the weight loss ratio of CS/MPP/C_3_S composite bone cement, which indicated that the degradation mechanism of bone cement was mainly the gradual dissolution of components. As is well known, Ca^2+^ and Mg^2+^ can promote the proliferation and differentiation of bone cells, and promote the synthesis of bone matrix, thus facilitating bone regeneration and healing. However, the high concentrations of Ca^2+^ and Mg^2+^ had inhibited effects on cells. Therefore, the slow release of Ca^2+^ and Mg^2+^ in bone cement not only maintained its promoting effect on new bone formation, but also avoided adverse effects on bone repair caused by high concentrations. In addition, the release mechanism of Mg^2+^ and Ca^2+^ was explained using the Korsmeyer–Peppas kinetic model. The simulation curves and equations are shown in Figure 4c and Figure 4d, respectively. In the simulation equations of Mg^2+^ and Ca^2+^, the *n* values were 0.28 and 0.45, respectively (*n* is the characteristic parameter of the release mechanism). For cylindrical samples such as bone cement, when *n* ≤ 0.45, the release mechanism of Mg^2+^ and Ca^2+^ is Fickian diffusion, which is affected by the diffusion of water in the matrix, matrix erosion, and matrix swelling [42].

### 3.4. Cytotoxicity and Osteogenic Differentiation In Vitro

**Figure 5:**
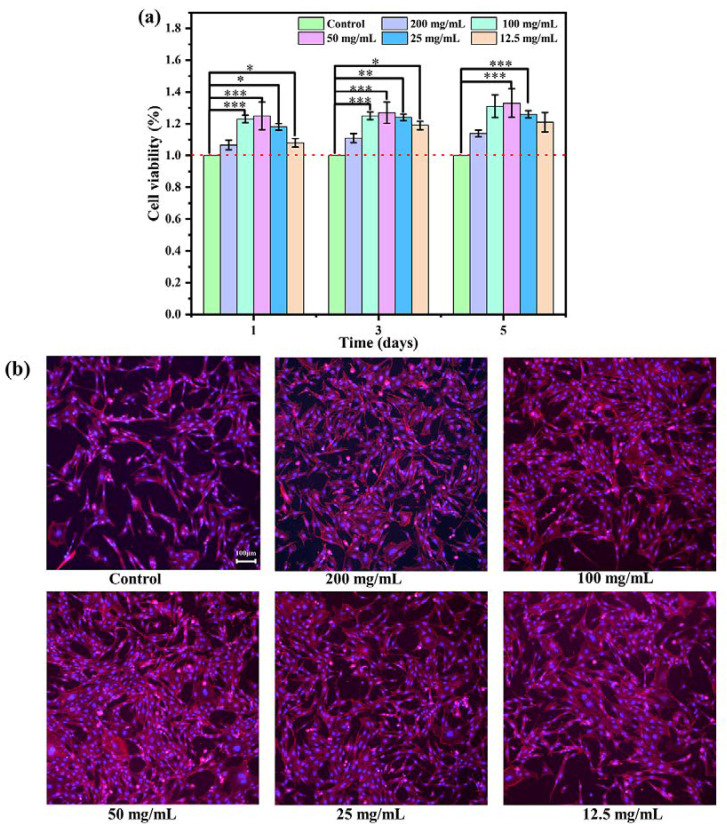
Cytotoxicity of CS/MPP/C_3_S composite bone cement. (**a**) Cell viability (%). (**b**) FIM images of the cytoskeleton (* *p* < 0.05, ** *p* < 0.01, and *** *p* ≤ 0.001).

The proliferation of MC3T3 cultured in different concentrations of bone cement extracts on days 1, 3, and 5 was evaluated using the CCK-8 assay kit. As shown in Figure 5a, the cell viability of each concentration exceeded 100% on days 1, 3, and 5, suggesting a promoting effect on cell proliferation. Actually, numerous studies showed the cell proliferation ability of CSC was inferior to that of the control group, likely attributed to acidic byproducts generated during its degradation [29,43,44,45,46,47]. It can be inferred that the biocompatibility of CS/MPP/C_3_S composite bone cement was greatly improved. Note that the extraction with the original concentration (200 mg/mL) exhibited the lowest cell viability with 106%, 111%, and 114% on days 1, 3, and 5, respectively. This may be attributed to the highest concentration of Mg^2+^ in the original extraction solution. Although Mg^2+^ plays a crucial role in maintaining the normal function and vitality of osteoblasts, promoting bone tissue formation and repair, an excessive concentration of Mg^2+^ may adversely affect the vitality and function of osteoblasts. Additionally, cell morphology was assessed by staining the cytoskeleton. As shown in Figure 5b, all cells exhibited polygonal shapes with obvious pseudopodia, indicating a healthy cell morphology. Compared with the cells in the control group, CS/MPP/C_3_S composite bone cement displayed a higher cell count and fuller cell morphology.

### 3.5. Osteogenic Differentiation

**Figure 6:**
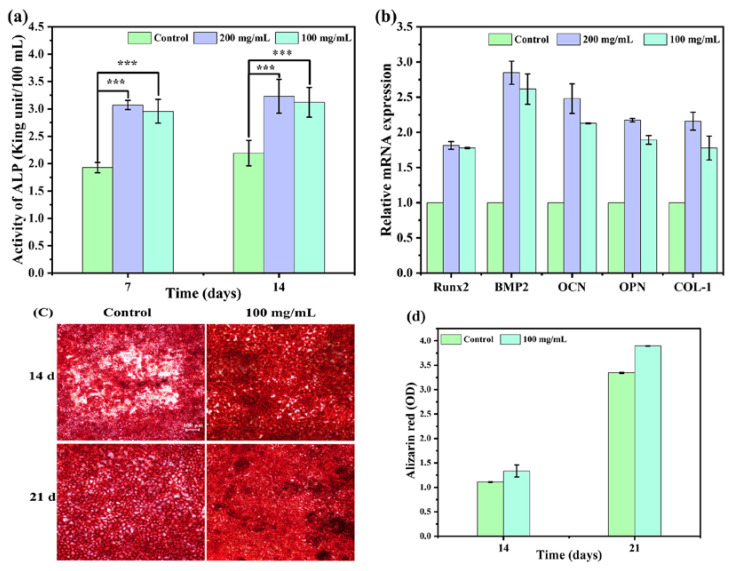
Osteogenic differentiation of CS/MPP/C_3_S composite bone cement. (**a**). ALP activity. (**b**) Expression of Runx2, BMP2, OCN, OPN, and COL-1 after 14 days of cell differentiation. (**c**) Alizarin Red staining on days 14 and 21 (the scale bar: 100 μm). (**d**) Semi-quantitative analysis of calcium nodules (*** *p* ≤ 0.001).

Bone formation is a complex process involving mesenchymal stem cell differentiation into osteoblasts, osteoblasts recruitment, initial bone mineral nuclei formation, and angiogenesis facilitating calcium ion influx for bone mineralization and calcification. It can be classified as a three-step process: (i) proliferation—increase in the number of pre-osteoblasts, (ii) differentiation—pre-osteoblasts become mature osteoblasts, and (iii) matrix mineralization—the formation of new bone matrix by the mature osteoblasts. ALP activity, a well-known osteoblastic marker, signifies early osteoblast genesis. The quantitative measurement of ALP could therefore reflect the level of osteoblast differentiation at an early stage. ALP activity in the culture supernatant on days 7 and 14 is shown in Figure 6a. Among them, the ALP activity of CS/MPP/C_3_S composite bone cement was significantly higher than that of the control group. The ALP activity of the 200 mg/mL extract on day 14 was 1.7 times higher than that of the control group. The slight increase in ALP activity between days 7 and 14 could be attributed to the role of ALP as an early marker of osteoblast differentiation, with its expression gradually declining as the cells progressed through the middle and late stages of differentiation. Moreover, the expression of osteogenesis-related genes was detected on day 14, which is shown in Figure 6b. BMPs play important roles in bone formation and bone cell differentiation by stimulating ALP activity and synthesis of proteoglycan, collagen type I (COL-1), fibronectin, and osteocalcin (OCN). The changing trends of osteogenesis-related gene expression were consistent with ALP activity. After 14 days, the expression levels of osteogenic-related genes (200 mg/mL), including Runx2, BMP2, OCN, OPN, and COL-1, were about 1.8, 2.8, 2.5, 2.2, and 2.2 times higher than those of the control group, respectively. Furthermore, calcium deposition is one of the markers of late osteogenic differentiation, and the degree of osteogenic differentiation could be characterized by observing the calcium deposition generated during the process of osteogenic differentiation. Calcium deposits were stained with Alizarin Red and semi-quantitative analysis was performed with hexadecylpyridinium chloride monohydrate. As shown in Figure 6c,d, both the control group and CS/MPP/C_3_S composite bone cement produced calcium nodules during cell differentiation. The red color in CS/MPP/C_3_S composite bone cement was significantly darker than that of the control group, and the red area on day 21 was significantly more than that on day 14. In addition, the results of the semi-quantitative analysis (Figure 6c) were consistent with the staining results. This further proved that CS/MPP/C_3_S composite bone cement had great potential to promote osteogenic differentiation.

In previous literature concerning the CSC [29,46,47,48,49], CSC could not form chemical bonds with bone tissue due to its poor biological activity in the early process of bone repair. Therefore, the modification or composite of CSC has become a research direction. In this study, CS/MPP/C_3_S composite bone cement had a higher ability to interact with mBMSCs, promoting osteogenesis and matrix calcification. Concretely, the ALP activity, the expression levels of osteogenic-related genes (Runx2, BMP2, OCN, OPN, and COL-1), and the calcium deposition of CS/MPP/C_3_S composite bone cement were higher than those of the control group during the process of osteogenic differentiation. This further demonstrated that the incorporation of MPP and C_3_S effectively improved the biological activity of CSC, providing ideas for further application of bone cement based on CS.

Researchers have adopted different improvement methods to address the shortcomings of short setting time, low compressive strength, poor injectability, fast degradation, and poor biological activity of CSC. For example, combinations of CSC and inorganic ceramic materials such as hydroxyapatite [50], calcium silicate [48], and bioglass [51] were tested. On the one hand, the longer solidification time, slower degradation, and alkalinity of these inorganic ceramic materials can be used to regulate the solidification time, degradation rate, and degradation microenvironment of CSC. On the other hand, these bioactive inorganic ceramic materials can also improve the biological activity of CSC and enhance the mechanical strength of CSC to some extent. Hao et al. [52] compared the absorption rates of CSC and CS/C_3_S composite bone cement implanted in rabbit femoral condylar defects. After 8 weeks, CSC completely degraded, while the CS/C_3_S composite bone cement remained (68.33 ± 3.69%), indicating that the combination of CS and C_3_S can effectively regulate the degradation rate of CSC. In addition, when organic polymers such as polylactic acid [53], gelatin [54], and carboxymethyl cellulose [55] were combined with CSC, the mechanical properties and operability of CSC were improved. For example, Lewis et al. [56] added organic polymer carboxymethyl cellulose to CSC, effectively improving its mechanical properties and operability, but this accelerated the degradation of bone cement. This study added MPP, C_3_S, and HPMC to CSC, aiming to regulate the degradation rate and microenvironment of CSC through C_3_S, improve the injectability and mechanical properties of CSC through HPMC, and enhance the biological activity and osteogenic ability of CSC through the bioactive ingredient MPP. The results indicated that CS/MPP/C_3_S composite bone cement had suitable solidification time (15 min), injectability (93%), compressive strength (26 MPa), degradation rate (35% within 8 weeks), as well as good biocompatibility and osteogenic activity, providing a novel and useful formulation of bone cement.

## 4. Conclusions

In order to address the limitations associated with rapid solidifying, low mechanical strength, and poor biological activity of CSC, we developed a composite bone cement by introducing MPP, C_3_S, and HPMC to the CSC. The CS/MPP/C_3_S composite bone cement exhibited excellent performance compared to CSC. In terms of operability, the setting time was extended from 5 min to 15 min, the injectability was increased from 34% to 93%, and the compressive strength was enhanced from 14 MPa to 26 MPa. In terms of degradability, the degradation rate was slowed down to 35% in 8 weeks, the degradation mechanism was mainly the dissolution of components, and the ions were released by diffusion. Furthermore, CS/MPP/C_3_S composite bone cement exhibited favorable biocompatibility and osteogenic activity. The cell proliferation rates were higher than the control at any concentration extracts, and at any time. Even 200 mg/mL extract reached 114% after 5 days. Moreover, during the process of cell differentiation, the expression of osteogenic-related genes, including Runx2, BMP2, OCN, OPN, and COL-1, in the 200 mg/mL extract was 1.8, 2.8, 2.5, 2.2, and 2.2 times higher than those of the control, while ALP activity and calcium nodule were also higher than those of the control. Consequently, this innovative CS/MPP/C_3_S composite bone cement is a prospective candidate for bone repair materials.

## Figures and Tables

**Table 1:** Primer sequences of q-PCR.

Genes	ID	Direction	Sequences
β-Actin	11461	FORWARD	GTGCTATGTTGCTCTAGACTTCG
		REVERSE	ATGCCACAGGATTCCATACC
runx2	12393	FORWARD	ATGGCGTCAAACAGCCTCTTC
		REVERSE	TGGTGCTCGGATCCCAAAAG
BMP2	12156	FORWARD	TCTTCCGGGAACAGATACAGG
		REVERSE	TGGTGTCCAATAGTCTGGTCA
OCN	12096	FORWARD	AAGACCGCCTACAAACGCATCTAT
		REVERSE	GCACTTCCTCATCTGAACTTTATTTTG
OPN	20750	FORWARD	TTGGTGACTTGGTGGTGATCTAGT
		REVERSE	TCTCCTCTGAGCTGCCAGAATC
COL1	12842	FORWARD	TAAGGGTCCCCAATGGTGAGA
		REVERSE	GGGTCCCTCGACTCCTACAT

## Data Availability

Data are contained within the article.

## Supplementary Materials

The following supporting information can be downloaded at: https://www.mdpi.com/article/10.3390/ma17081861/s1, Table S1: List of abbreviations; Table S2: The compressive strength of bone cement with different plasticizers.
