# Supplementary material for: A Bioactive Degradable Composite Bone Cement Based on Calcium Sulfate and Magnesium Polyphosphate
Source: Materials (Basel). 2024 Apr 18;17(8):1861. doi: 10.3390/ma17081861 (PMC11051185; doi:10.3390/ma17081861)
Supplement: Supplementary file 1 [file materials-17-01861-s001.zip › materials-2943327-supplementary.pdf]

Supporting Information

A bioactive degradable composite bone cement based on calcium sulfate and magnesium polyphosphate

Suping Peng[1], Xinyue Yang[1], Wangcai Zou[1], Xiaolu Chen[2], Hao Deng[2], Yonggang Yan[2] * Qiyi Zhang[1] *,

[1]School of Chemical Engineering, Sichuan University, Chengdu, Sichuan, 610065, P. R. China; 2021223075215@stu.scu.edu.cn (Suping Peng)

[2]College of Physics, Sichuan University, Chengdu, Sichuan, 610065, P. R. China

[*]Correspondence: qyzhang-scu@163.com (Qiyi Zhang); yan_yonggang@vip.163.com (Yonggang Yan)

Table S1. List of abbreviations

Full name	Abbreviation
Calcium sulfate bone cement	CSC
Magnesium polyphosphate	MPP
Calcium sulfate	CS
Tricalcium silicate	C_3S
Hydroxypropyl methylcellulose	HPMC
α-calcium sulfate hemihydrate	α-CSH
Calcium sulfate dihydrate	CSD
Alkaline phosphatase activity	ALP
Cell Counting Kit-8	CCK-8
Polytetrafluoroethylene	PTFE
Minimum Essential Medium alpha	MEM-α
Fetal bovine serum	FBS
Mouse embryonic osteoblasts cells	MC3T3
4',6-diamidino-2-phenylindole dihydrochloride	DAPI
Mouse bone marrow-derived mesenchymal stem cells	mBMSCs
Dulbecco's modified Eagle's medium	DMEM
Carboxymethyl chitosan	CMCS
Carboxymethyl cellulose sodium	CMC-Na

Full name	Abbreviation
Chitosan quaternary ammonium salt	HACC
Pullulan polysaccharide	Pul
Hyaluronic acid	HA
Hydroxyethyl cellulose	HEC
Chitosan oligosaccharides	COS

Table S2. The compressive strength of bone cement with different plasticizers

Plasticizers (1wt %)	CS (g)	MPP (g)	C_3S (g)	Compressive strength (MPa)
CMCS	4.35	0.5	0.15	6.3±0.5
CMC-Na	4.35	0.5	0.15	7.1±0.8
HACC	4.35	0.5	0.15	9.3±0.6
Pul	4.35	0.5	0.15	8.3±0.8
HA	4.35	0.5	0.15	11.3±0.6
HPMC	4.35	0.5	0.15	20.2±2.3
HEC	4.35	0.5	0.15	7.4±0
COS	4.35	0.5	0.15	5.4±1.4
